# Electrons receive individual treatment with electron-event representation

**DOI:** 10.1107/S2052252520011616

**Published:** 2020-08-29

**Authors:** Radostin Danev

**Affiliations:** a Graduate School of Medicine, University of Tokyo, N415, 7-3-1 Hongo, Bunkyo-ku 113-0033 Tokyo, Japan

**Keywords:** cryo-EM, direct electron detectors, image format

## Abstract

Electron-event representation saves the spatial and temporal coordinates of every electron with ultimate accuracy. Through maximum signal extraction, it will improve the performance of electron cryo-microscopy.

Up until a decade ago, photographic film provided the best performance for the recording of cryo-electron microscopy (cryo-EM) images. Digital cameras using a phosphor scintillator have also been available since the late 1990s, but despite being more convenient to use their signal-to-noise characteristics were significantly inferior to those of film. Irrespective of their differences, both recording methods were far from the theoretically achievable performance and limited the typical resolution of cryo-EM reconstructions to a ‘blobology’ (~10 Å) level. The inadequacy of these legacy image capture methods was resolved around 2012 with the first commercial introduction of direct electron detectors (DEDs). Together with substantial advances in image processing algorithms, these new cameras played a fundamental role in the great leap in cryo-EM performance.

As the name suggests, DEDs detect electrons through their direct interaction with the active silicon layer of the camera chip (McMullan *et al.*, 2016 ▸). They are based on CMOS technology that gives them radiation resistance and fast readout speeds. Unlike previous methods that recorded a single integrated image, DEDs are read out continuously during the exposure with a frame rate of a few hundred frames per second. Each frame contains a small number of incident electron events that are sufficiently dispersed to allow on-the-fly numerical identification and localization of individual electrons. The process is known as ‘electron counting’ and virtually eliminates detection noise, resulting in a superior signal-to-noise ratio. Furthermore, the localization of electron events can be performed with sub-pixel accuracy to extract information beyond the physical Nyquist frequency of the detector. The current approach for recording of the counted electron data is to accumulate the counts from several frames on an image grid and store it as an ‘exposure fraction’ in a movie. The pixel dimensions of the fraction are either the same as the detector or two-times oversampled for the so-called ‘super-resolution’ mode. The number of detector frames that are accumulated into each fraction is a parameter that must be decided before the experiment and cannot be changed after the data have been collected. Smaller fractions and super-resolution sampling can produce higher quality results but also significantly increase the size of the recorded movies. Therefore, researchers must make a compromise by selecting recording parameters that will provide close to optimal results without requiring excessive data storage space. In some unfortunate cases, after processing the data the a priori parameter choice may turn out to have been suboptimal, but apart from performing another experiment there is no way to correct it.

In their paper in this issue of **IUCrJ**, Guo and colleagues (Guo *et al.*, 2020 ▸) present a new data recording and storage format for direct electron detectors called ‘electron-event representation’ (EER). Instead of recording movies, it captures each individual electron event and saves its coordinates in (*x*, *y*, time) space (Fig. 1 ▸). Consequently, the EER method preserves the spatial and temporal information for every electron and resolves completely the compromise between information retention and data size that is currently imposed by the movie recording format. Furthermore, EER uses fourfold spatial super-sampling which allows it to capture high-resolution image components beyond the physical Nyquist periodicity of the detector, with sufficient signal-to-noise ratio to be usable in cryo-EM reconstructions. The authors provide an experimental demonstration with an apoferritin test sample that has reached a resolution 1.2 times the Nyquist frequency. Having good information preservation up to and even beyond Nyquist will allow researchers to comfortably collect data at lower microscope magnifications that give a larger sample area field of view, without high-resolution signal loss. This will increase the number of particles per image or the captured cellular area in cryo-tomography and boost the experimental throughput. The complete preservation of temporal information will also improve the performance by allowing finer sampling and interpolation on the single frame level during the beam-induced motion correction step of data processing. This will provide means for recovering a much larger portion of the high-resolution information that is present only in the very beginning of the exposure, before radiation damage of the sample has progressed, but also when the majority of beam-induced motion of the specimen is taking place. Because the EER format contains a complete representation of the electron events, experimentation and optimization of the motion correction fractionation parameters can be performed freely during data processing.

EER is another significant step in the development of cryo-EM detector technology. It removes experimental restrictions and improves detector performance while also freeing researchers from mandatory data reduction decisions that had to be made with previous recording methods.

## Figures and Tables

**Figure 1:**
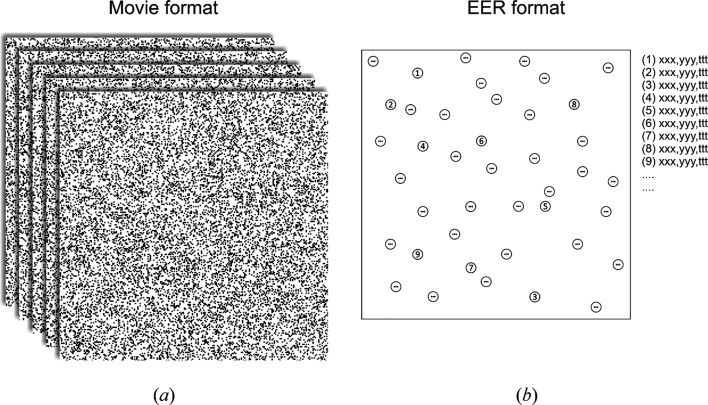
Illustrative comparison of (*a*) the current movie recording format and (*b*) the newly developed electron-event representation (EER) format. In the movie format, electron counts are accumulated in fractions of predefined time length that are stored as an image stack. The fraction length must be decided before the experiment and determines the shortest time interval at which beam-induced motion correction can be performed during data processing. Shorter fractions allow finer sampling but also increase the movie file size, necessitating a practical compromise with the fraction length. In the EER format, each electron is recorded with its spatial coordinates and the time of detection. The spatial coordinates have subpixel accuracy with fourfold super-sampling, which allows recovery of information beyond the physical Nyquist periodicity of the detector. During processing, the time interval for beam-induced motion calculations can be selected arbitrarily and optimized for best performance.
